# Emergence of nontoxic mutants as revealed by single filament analysis in bloom-forming cyanobacteria of the genus *Planktothrix*

**DOI:** 10.1186/s12866-016-0639-1

**Published:** 2016-02-25

**Authors:** Qin Chen, Guntram Christiansen, Li Deng, Rainer Kurmayer

**Affiliations:** College of Natural Resources and Environment, Northwest A & F University, Taicheng Road 3, 712100 Yangling, Shaanxi Province P. R. China; Research Institute for Limnology, University of Innsbruck, Mondseestrasse 9, 5310 Mondsee, Austria; Helmholtz Zentrum München, Institute of Groundwater Ecology, Ingolstädter Landstrasse 1, 85764 Neuherberg, Germany

**Keywords:** cyanoHABs, Microcystin, Microevolution, Mobile elements, Single-colony PCR, *in situ* observation

## Abstract

**Background:**

Bloom-forming cyanobacteria cause toxic algae outbreaks in lakes and reservoirs. We aimed to explore and quantify mutation events occurring within the large *mcy* gene cluster (55 kbp) encoding microcystin (MC) biosynthesis that inactivate MC net production. For this purpose we developed a workflow to detect mutations *in situ* occurring anywhere within the large *mcy* gene cluster as amplified from one single filament of the red-pigmented cyanobacterium *Planktothrix rubescens*. From five lakes of the Alps eight hundred *Planktothrix* filaments were isolated and each individual filament was analyzed for mutations affecting the *mcy* genes.

**Results:**

Mutations inactivating MC synthesis were either through an insertion element ISPlr1 or the partial deletion of *mcy* genes. Neutral mutations not affecting MC biosynthesis occurred within two intergenic spacer regions, either through the insertion of a Holliday-junction resolvase RusA or ISPlr1. Altogether, the insertions affected a few *mcy* genes only and their location was correlated with regions similar to repetitive extragenic palindromic DNA sequences (REPs). Taking all of the filaments together, the mutations leading to the inactivation of MC synthesis were more rare (0.5–6.9 %), when compared with the neutral mutations (7.5–20.6 %). On a spatial-temporal scale the ratio of MC synthesis-inactivating vs. neutral mutations was variable, e.g., the filament abundance carrying partial deletion of *mcy*D (5.2–19.4 %) and/or *mcy*HA (0–7.3 %) exceeded the abundance of neutral mutations.

**Conclusions:**

It is concluded that insertion events occurring within the *Planktothrix mcy* gene cluster are predictable due to their correlation with REPs. The frequency of occurrence of the REPs within the *mcy* gene cluster of *Planktothrix* relates to the rather common mutation of *mcy* genes in *Planktothrix*. Spatial-temporal variable conditions may favor the emergence of partial *mcy* deletion mutants in *Planktothrix*, in particular a higher proportion of genotypes resulting in inactivation of MC synthesis might be caused by increased ISPlr1 activity.

**Electronic supplementary material:**

The online version of this article (doi:10.1186/s12866-016-0639-1) contains supplementary material, which is available to authorized users.

## Background

The filamentous, bloom-forming cyanobacterium *Planktothrix*, occurs in the pelagic zone of lakes and reservoirs, and is one of the main producers of microcystin (MC) – a hepatotoxin that poses a health threat to humans and livestock [1]. The blooming of *Planktothrix* regularly leads to toxic algae outbreaks, e.g., in December 2013 the water distributed in Užice in Serbia was banned for drinking and food preparation affecting thousands of people because of the intense bloom of toxic *P. rubescens* in Lake Vrutci, which served as the source of water supply. There is a documented history on these relatively sudden mass appearances of toxic *P. rubescens*, particularly in reservoirs [2–4], which is of relevance not at least due to their ongoing construction [5].

MC is synthesized by a nonribosomal peptide synthetase (NRPS) encoded by the *mcy* gene cluster containing nine to ten genes that have been elucidated from three abundant MC-producing genera *Microcystis*, *Planktothrix* and *Anabaena* [6–8]. *Planktothrix* [9] and *Anabaena* [10] have been reported to contain mutations within the *mcy* gene cluster. In *Planktothrix* the *mcy* gene cluster is affected by (i) recombination events affecting enzymatic domains [11, 12], and (ii) by inactivation, e.g., due to the partial deletion of *mcy* genes [13] or the insertion of transposable elements [9]. The latter mutations that lead to nontoxic subpopulations have been found to co-occur with the toxic subpopulation in nature [13, 14]. Individuals carrying mutations inactivating the *mcy* genes could not be differentiated from those carrying the original *mcy* gene cluster by phylogenetic analysis using housekeeping genes [15, 16]. This result implies that the respective genotypes carrying those mutations in the *mcy* gene cluster are evolutionary relatively young and/or possibly the selective pressure is not high enough to favor their phylogenetic fixation. Previously, the specific mutant *mcy* genotypes were found to be distributed among twelve lakes of the Alps (Austria, Germany, Switzerland) and to occur consistently irrespective of the total population density [14]. Only recently, through an almost 30-year observation period, a gradual increase of one *mcy* mutant genotype carrying a 1.8 kbp deletion of the *mcy* gene cluster was discovered [17].

Currently, our understanding on the factors leading to the mutations within the *mcy* gene cluster is low, both with regard to molecular factors as well as the ecological factors influencing the activity of mutagenic elements such as transposases. In general, physiological stress conditions have been observed to favor transposase activity [18]. Since mutations often lead to the inactivation of MC synthesis, the emergence of nontoxic genotypes in *Planktothrix* populations would decrease the MC production, which is potentially relevant since *P. rubescens* populations have been described with the on average highest MC content in nature [19].

We aimed to develop a workflow to detect mutations *in situ* occurring within the large *mcy* gene cluster as amplified from one single *P. rubescens* filament. Such results can pave the way to understand the regulation of the occurrence of mutations, especially those inactivating MC synthesis. In addition several mutations within the *mcy* gene cluster have been characterized previously using isolated clonal strains thus, enabling to differentiate between mutations resulting in inactivation of MC synthesis and neutral mutations not affecting MC synthesis [9, 11, 13, 14]. Since the entire *mcy* gene cluster spans > 55,000 bp, a sustainable technique is required that is able to amplify fragments covering the entire *mcy* gene cluster. This would make it possible to analyze (i) a population for all mutations affecting MC synthesis in real-time, (ii) the phylogenetic divergence of those mutants when compared with genotypes still containing the original *mcy* gene cluster using housekeeping genes, (iii) the abundance of each of the discovered *mcy* mutants on a spatial and temporal scale.

## Results

### Influence of filament length on PCR results

**Fig. 1:**
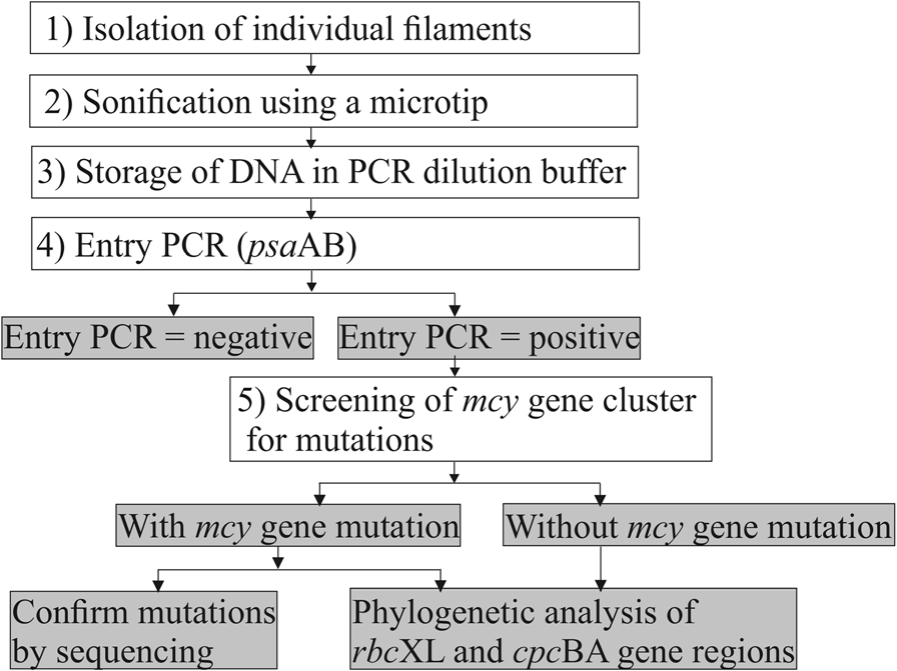
Flow diagram showing steps of *Planktothrix rubescens* filament isolation and analysis (white boxes) and obtained results (grey boxes). For PCR conditions and primers see text and Additional file 6

In total, 914 filaments of red-pigmented *P. rubescens* were isolated from five lakes in the Alps (Table 1) and analyzed for mutations affecting the *mcy* genes (Fig. 1). Taking together all filaments, 87.5 % (800 out of 914) showed a PCR product when amplifying the intergenic spacer region (IGS) between *psa*A and *psa*B. A minimum of hundred positive filaments from each of the five populations were sampled.

**Table 1:** Number of isolated *Planktothrix rubescens* filaments, percentage of PCR-positive filaments, and percentage of mutations

	Filament number	Positive Entry PCR (%)	Characteristics of filaments		Mutations within the *mcy* gene cluster (%)						
			Average (min, max) length (μm)	Average (min, max) cell number^a^	MC synthesis not inactivated			Inactivation of MC synthesis			
					*mcy*TD insertion	*mcy*EG insertion	Short *mcy*A variant	*mcy*D insertion	*mcy*A insertion	*mcy*D deletion	*mcy*HA deletion
Mondsee (AT)											
Mar 2012	105	95.2	1356 (484, 2574)	435 (155, 825)	5.7	3.4	81.1	0	0	5.2	0
Jun 2012	108	92.6	1251 (506, 2222)	401 (162, 712)	2.0	2.0	67.7	2.0	1.0	7.2	3.0
Sep 2012	102	98	1653 (990, 2618)	530 (317, 839)	27.0	1.0	44.8	0	0	19.4	7.3
Apr 2013	107	93.4	1392 (748, 2354)	446 (240, 754)	13.1	0	53.3	1.0	0	18.0	7.1
Other lakes											
Wörthersee (AT)	136	73.5	2200 (858, 6600)	705 (275, 2115)	18.4	36.0	67.3	1.0	4.0	5.0	3.1
Zürichsee (CH)	121	82.6	1032 (440, 1738)	331 (141, 557)	25.3	9.0	45.0	0	0	1.0	1.0
Hallwilersee (CH)	120	83.3	1555 (704, 2772)	498 (226, 888)	42.6	5.3	82.4	0	0	0	0
Ammersee (DE)	115	87	1444 (638, 2420)	463 (204, 775)	37.6	4.2	60.6	0	0	0	0
Total	914	87.5	1479 (440, 6600)	474 (141, 2115)	20.6	7.5	59	0.5	0.6	6.9	2.6

^a^estimated from linear regression curve (Additional file 1)

Due to the observed variability in filament length (Table 1) it was important to know whether the shorter filaments contained sufficient DNA template for PCR. The minimum filament length (88 μm) that was recorded corresponded to 29 cells (Additional file 1). Taking all filaments together, the filaments that were found PCR positive differed marginally in length (average 477 ± 7 SE) from the filaments that were found PCR negative (447 ± 21), (Mann–Whitney Rank Sum Test, *p* = 0.04, Student’s *t*-test, *p* = 0.17, Additional file 1). When comparing the proportion of PCR positive filaments between populations rather the opposite was observed, i.e., the on average longest filaments isolated from Lake Wörthersee showed the lowest proportion of PCR positive samples (73.5 %). Thus the influence of DNA template amount on PCR result variability between populations was considered of minor importance.

### Mutations within the microcystin synthesis gene cluster

**Fig. 2:**
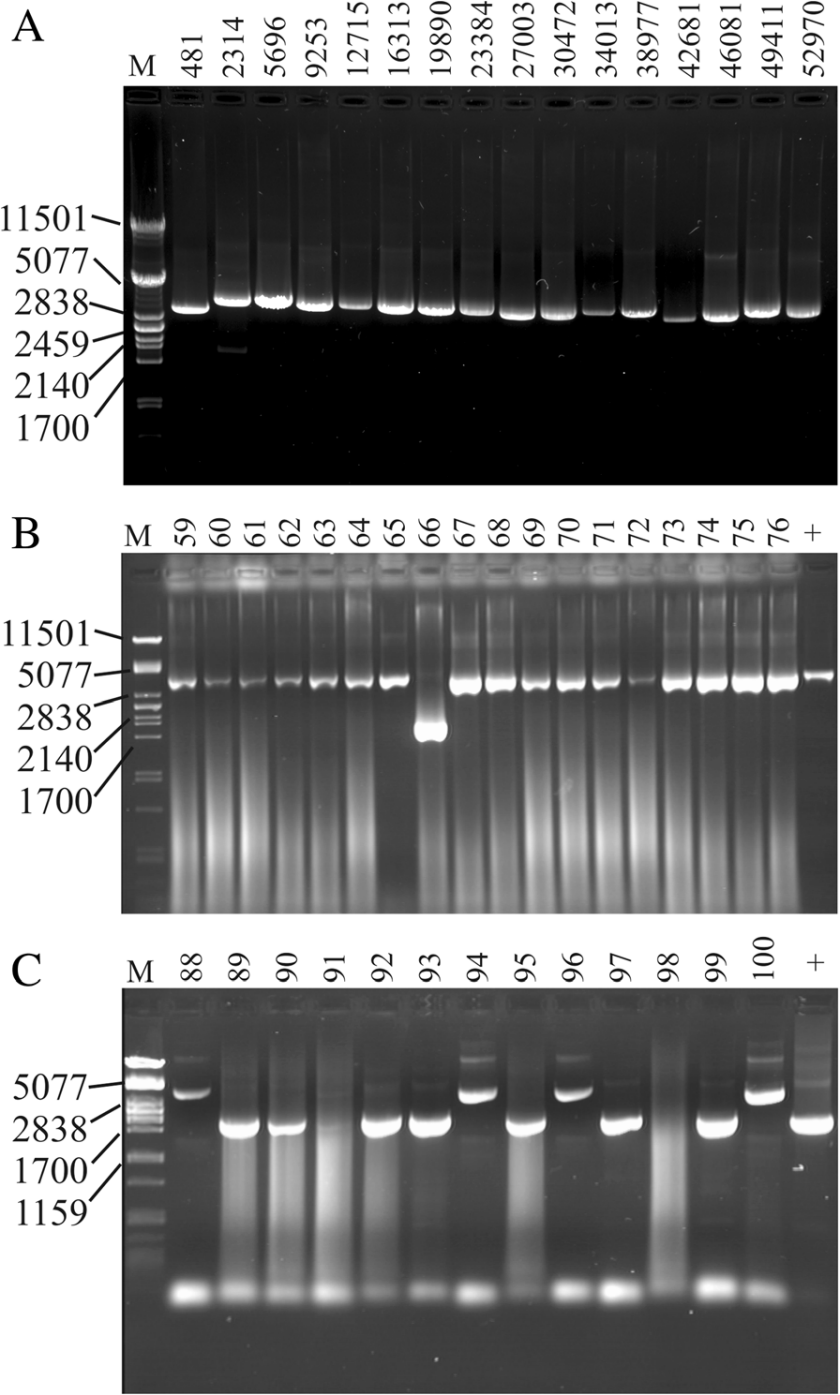
PCR amplification of the entire *mcy* gene cluster from single *Planktothrix rubescens* filaments to detect mutations by PCR size polymorphism. **a** Amplification of DNA fragments (approx. 3 kb) of the entire *mcy* gene cluster from DNA isolated from one individual filament (No 11, Mondsee, 15 Mar 2012). The nucleotide pos. represents the binding position of the forward primer according to the sequence of the *mcy* gene cluster from *P. agardhii* NIVA-CYA126/8 (AJ441056), (Additional file 6). **b** Amplification of DNA fragment using primer pair F*mcy*2+/− from individual filaments No 59–76 isolated from Zürichsee (20 Jun 2012). Filament No 66 shows a deletion in *mcy*D. **c** Amplification of DNA fragment using primer pair F*mcy*8+/F*mcy*m8- (1.75 kb) from individual filaments No 88–100 isolated from Wörthersee (3 Apr 2012). The larger PCR products (Filament No 88, 94, 96, 100) indicate the insertion into the IGS region (1423 bp) between *mcy*E and *mcy*G. M, PstI lambda DNA size marker. Positive control (+) was amplified from *P. agardhii* NIVA-CYA126/8 (AJ441056)

All *Planktothrix* filaments that were found to be PCR positive also contained the *mcy* gene cluster. An example for PCR amplification of the entire *mcy* gene cluster from one single filament is shown in Fig. 2a. Sixteen primer pairs were used to amplify *mcy* gene fragments of 3.5 kbp without interruption.

Among all of the filaments, 20 % were indistinguishable from the reference *mcy* gene cluster described from *P. agardhii* NIVA-CYA126/8 (Access No. AJ441056), while 80 % showed polymorphisms in PCR product size (Fig. 2b, c). Among these, one smaller PCR product (59 %) was due to the functional recombination of the *mcy*AA1 adenylation domain, i.e., the replacement of the *mcy*A variant containing a gene of the N-methyltransferase at pos. 34,656 – 37,592 (AJ441056) by the shorter *mcy*A variant lacking the N-methyltransferase as described [11].

**Fig. 3:**
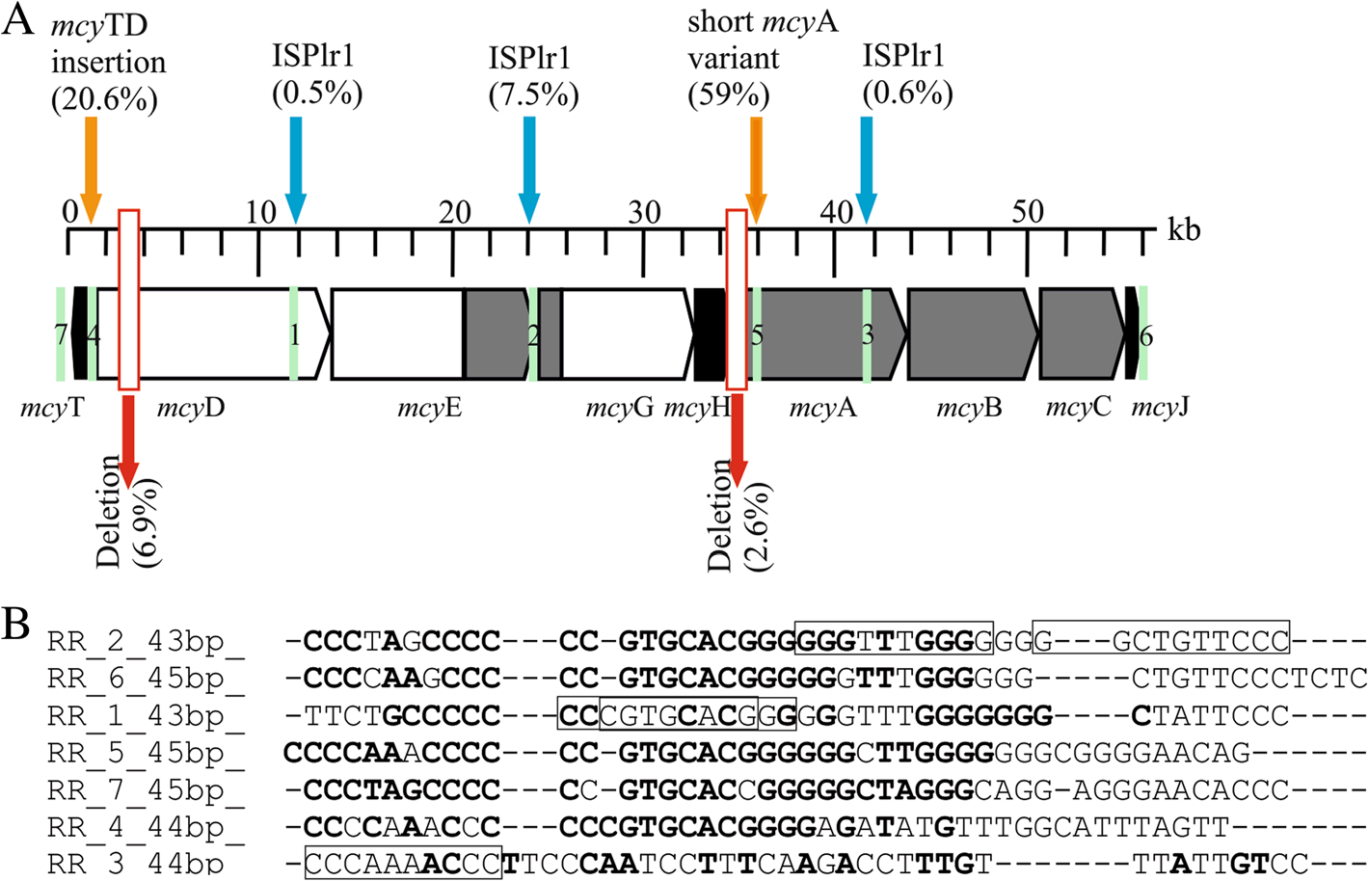
**a** Schematic view of *Planktothrix mcy* gene cluster and location of mutations found in individual filaments and location of repetitive regions 1 – 7 (in green). Taking all filaments together the relative frequency of each mutation is given in parentheses. ISPlr1, *P. rubescens* IS element containing the conserved DDE domain for DNA transposition [9]; **b** Alignment of repetitive sequence regions within the *P. agardhii* NIVA-CYA126/8 *mcy* gene cluster (ASAK00000000). The framed boxes indicate the short directly repeated sequences (DR) of 10 bp in length resulting in insertion of transposable element ISPlr1. Bold letters indicate palindromic sequences

Four different polymorphisms with increased amplicon size were located within the IGS of *mcy*TD (20.6 %), within *mcy*D (0.5 %), within the IGS of *mcy*EG (7.5 %), and within *mcy*A (0.6 %), (Fig. 3a). The increase in amplicon size located between *mcy*T and *mcy*D (1194 bp) at pos. 1294 of the reference *mcy* gene cluster (AJ441056) was due to an inserted sequence coding for an ORF (pos. 997–742) homologous to crossover junction endodeoxyribonuclease RusA (85 aa, 100 % similarity on the amino acid level, *P. agardhii* NIVA-CYA15, WP_027250107), and a second ORF (pos. 742–58) which was a hypothetical protein distantly related to archaeal Holliday junction resolvase, (227 aa, 100 % similarity, *P. agardhii* NIVA-CYA15, WP_027250106). From the sequences obtained (*n* = 14) no variability of the insertion site could be found and only one orientation was observed. The other three insertions were caused by the IS element ISPlr1 (1423 bp), [14], i.e., within *mcy*D (at pos. 11,908 of the *mcy* gene cluster, AJ441056), within the IGS of *mcy*EG (at pos. 23,809), and within *mcy*A (at pos. 41,274), of which the insertion site and the orientation were described previously [9].

Deletions were detected within *mcy*D (6.9 %) and another deletion affected both *mcy*H and *mcy*A (2.6 %). The deletion within *mcy*D comprised 1665 bp (from pos. 2891 to 4555 of AJ441056), while the deletion occurring within *mcy*HA (1924 bp, from pos. 33,334 to 35,257 of the *mcy* gene cluster, AM990462.1) was described previously [9]. In summary, MC biosynthesis-inactivating mutations were observed only within a few genes (*mcy*D, H, A), while the other six genes part of the *mcy* gene cluster were not affected. For both housekeeping gene loci, *rbc*XL (seven genotypes, *n* = 64) and *cpc*BA (nine genotypes, *n* = 58), the one genotype representing the largest number of individuals (*rbc*XL, *n* = 50; *cpc*BA, *n* = 40) was comprised both of individuals without mutations or either carrying insertions (*mcy*D, *mcy*TD, *mcy*EG or *mcy*A) or partial deletions (*mcy*D and *mcy*HA), Table 2. Consequently, no phylogenetic diversification of specific *mcy* mutation genotypes was observed.

**Table 2:** Overview of sequences obtained from individual *P. rubescens* filaments during this study

Locus	Title	Length (bp)	Access No. (Isolated filaments^a^)
*mcy*TD	Crossover junction endodeoxyribonuclease RusA and putative Holliday junction resolvase	1194	KP315862 (*H22* ^b^); KP315863 (*A11* ^b^); KP315864 (*H11* ^b^, H31, *W22* ^b^, *Z14* ^b^, *Z24* ^b,e^, Z51^b^, *Z72* ^b^);
*rbc*LX	ribulose-1,5-bisphosphate carboxylase/oxygenase large subunit (*rbc*L) gene, and ribulose-1,5-bisphosphate carboxylase/oxygenase (*rbc*X) gene	383-390	KP315874 (*Z75*, *M78* ^b^, *W22* ^b^, A10^b^); KP315875 (M41^c^) ;KP315876 (*H39*); KP315877 (A20^b^); KP315878 (*M27*, *H81* ^b^, *H96*, *W36* ^d,e^, *Z66* ^c,f^); KP315879 (*A24*); KP315880 (*M2*, M7^e^, M19^c^, M21^e^, *M40*, M49^c^, *M52*, *M65* ^b^, *M80* ^b^, M85^e^, *W3*, *W4*, W13^e^, *W17*, *W29*, W35^b^, *W39* ^e^, *W53*, *W82* ^e^, Z4, Z5^e^, Z7^b^, *Z14* ^b^, *Z18*, *Z24* ^b,e^, *Z52*, Z59^e^, *Z96*, *H19*, *H20*, H31^b^, *H32*, H49^e^, H84, H95^e^, A11^b^, *A13*, A18^e^, A30^b^, *A32*, *A45*, *W11* ^g^, W18^b,g^, *W23* ^b,f^, *W34* ^b,f^, *W40* ^c^, *W47* ^c,f,g^, W63^b,g^, *W81* ^c^, W87^c^, *W93* ^c^);
*cpc*BA	partial *cpc*B gene for phycocyanin beta subunit and partial *cpc*A gene for phycocyanin alpha subunit	470	KP315865 (*M2*, *M52*, *Z4*, *Z18*, H96, H49^e^, *A32*); KP315866 (M21^e^); KP315867 (*W29*); KP315868 (W18^b,g^); KP315869 (*W4*); KP315870 (*M27*, *M40*, M85^e^, *W3*, *W53*, *Z52*, *Z96*, *H19*, *H20*, *H32*, *H39*, *A13*, *A24*, *A45*, M7^e^, *W39* ^e^, W13^e^, *W82* ^e^, Z5^e^, Z7^b^, *Z14* ^b^, *Z24* ^b,e^, Z59^e^, H31^b^, *H81* ^b^, *H94* ^b^, H95^e^, *A10* ^b^, A11^b^, A18^e^, A30^b^, M19^c^, M41^c^, M49^c^, *W11* ^g^, *W40* ^c^, *W81* ^c^, W87^c^, *W93* ^c^, *Z66* ^c,f^, H84); KP315871 (*A34*); KP315872 (*M65* ^b^, *M78* ^b^, W35^b^, *W23* ^b,f^, W63^b,g^); KP315873 (M74^c^);
*mcy*D	partially deleted *mcy*D gene of the MC synthetase	1964	KP710231 (M14^c^, M19^c^, M41^c^, M74^c^, *W40* ^c^, *W47* ^c,f,g^, *W49* ^e^, *W81* ^c^, W87^c^, *W93* ^c^, *Z66* ^c,f^)
*mcy*A	partial *mcy*A gene of MC synthetase, short *mcy*AA1 variant, 5′end + 3′end of recombination site	447, 428	KP315861 (*A16*, *A33*, *H9*, *H59*, *MNewSp6* ^b^, *MNewSp92*, *W29*, *W98* ^e^, *Z21* ^b^, *Z94*)

^a^The recombination of the short *mcy*A variant was indicated by italic font (M19, M41 and M74 was unkown), *A* Ammersee, *H* Hallwilersee, *M* Mondsee (March 2012), *W* Wörthersee, *Z* Zürichsee, *MNewSp* Mondsee (Apr 2013)

^b^filament carrying *mcy*TD insertion; ^c^ *mcy*D deletion; ^d^ *mcy*D insertion; ^f^ *mcy*EG insertion; ^e^ *mcy*A insertion; ^g^ *mcy*HA deletion

### Direct repeats and repetitive sequences within mutations

The sequencing of the ISPlr1 elements inserted into the *mcy* gene cluster (*n* = 17) revealed identical inverted repeats (IRL: 5′-CAGGGCTGTTTCA -3′ and IRR: 5′-TGAAACAGCCCTG-3′). The ISPlr1 elements also showed similarity with regard to the direct repeat (DR) sequence (10 bp). In total, five DR sequences were found, i.e., for the *mcy*D gene (5′-CCCGTGCACG-3′ or 5′-CGTGCACGGG-3′), for the *mcy*EG-IGS region (5′-GGGTTTGGGG-3′ or 5′-GGCTGTTCCC-3′), and for the *mcy*A gene (5′-CCCAAAACCC-3′). Within the entire reference *mcy* gene cluster of *P. agardhii* NIVA-CYA126/8 (AJ441056), seven GC rich repetitive regions (RRs), which were 43–45 bp in length, were identified. These RRs contained the above mentioned DR sequences and were similar to the repetitive extragenic palindromic DNA sequences (REPs) described in Proteobacteria and Actinobacteria [20] and were predicted to form DNA stem-loop hybridization [21]. RR 1 (within *mcy*D), RR 2 (within *mcy*EG-IGS), and RR 3 (within *mcy*A), all showed insertion by ISPlr1. RR 4 was located at the 5′end of the *mcy*TD insertion (the putative resolvase), while RR 5 was located within the recombination leading to the short *mcy*A variant (Fig. 3). RR 6 was found 121 bp downstream of *mcy*J, and RR 7 was located 622 bp upstream of *mcy*T. For RR 6 no mutation was observed (Additional file 2). The same IRR sequence of ISPlr1 (5′-TGAAACAGCCCTG-3′) was also observed next to RR 7. In summary, within the *Planktothrix mcy* gene cluster the insertion of ISPlr1 elements was not randomly distributed but correlated with repetitive DNA described as REPs from bacteria in general.

### Abundance of mutations

One fifth of the population (*n* = 162, 20 %) did not show any detectable mutation in comparison with the reference *mcy* gene cluster (AJ441056). Most frequently, the filaments contained one mutation (*n* = 492, 62 %), while filaments carrying two mutations (*n* = 142, 18 %) or three mutations (*n* = 4, 0.5 %) were rare. In general, the recombination resulting in the short *mcy*A variant constituted the dominant part of the population (45 - 82 %, Table 1). The second most abundant filament number was found to contain the *mcy*TD insertion (2 - 43 %), while 0 - 36 % carried the *mcy*EG insertion. Within IS element caused insertions, the number of ISPlr1 elements inserted into the IGS of *mcy*EG exceeded the number of ISPlr1 elements inserted into *mcy*D or *mcy*A considerably. Taking all filaments together, the mutations leading to the inactivation of MC synthesis were more rare (0 - 7 %), when compared with the abundance of the MC synthesis neutral mutations (7.5 - 20.6 %).

**Fig. 4:**
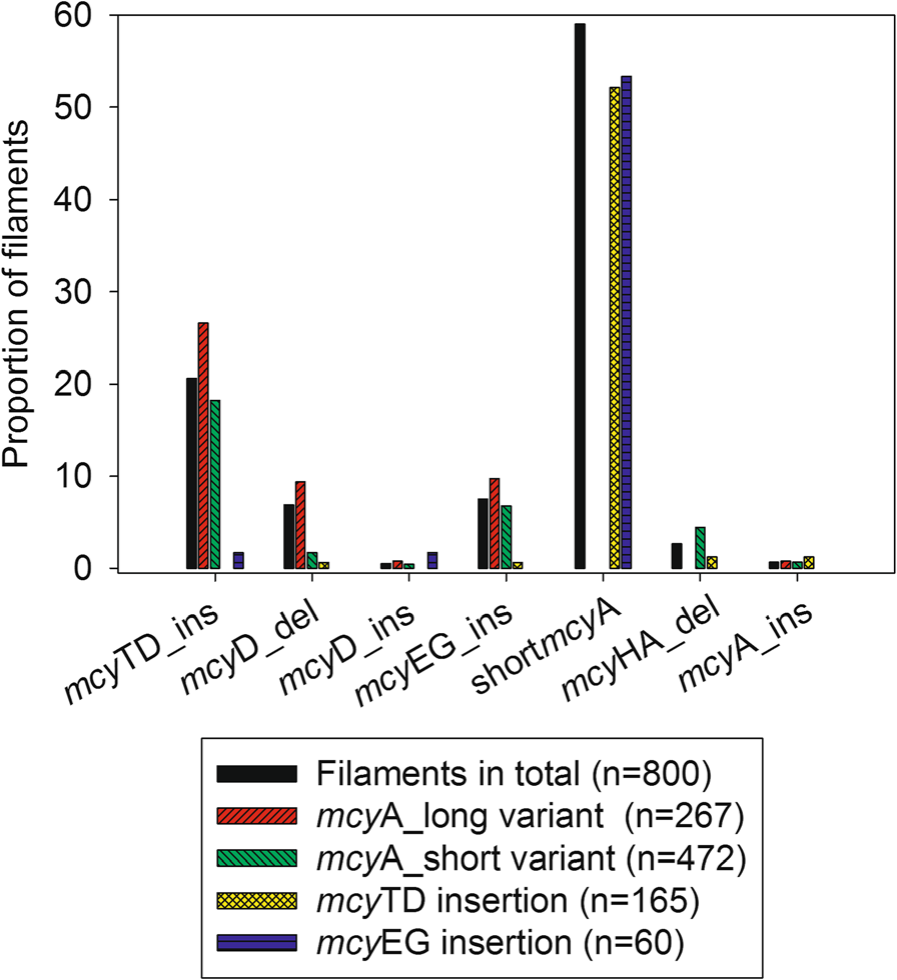
Proportion of *mcy* gene cluster mutations found in the total *Planktothrix* population and in subpopulations. Relative frequency of various *mcy* gene cluster mutations in relation to the number of total filaments, the filaments carrying the long or short *mcy*A variant, the presence of the putative resolvase (inserted into the *mcy*TD-IGS) and the ISPlr1 inserted into *mcy*EG-IGS

The filaments carrying either the long *mcy*A variant or the short *mcy*A variant contained a rather similar proportion of all the other mutations (Fig. 4). Only the *mcy*HA deletion (0 - 7 %) was perfectly linked to the *mcy*A short variant genotype. Filaments carrying a deletion in *mcy*HA (*n* = 21) also often had a deletion in *mcy*D (*n* = 15). In contrast, filaments carrying the *mcy*D partial deletion (*n* = 55) were comprised not only of *mcy*HA deletion, but also of undeleted *mcy*A short variant (*n* = 8) and long *mcy*A variant (*n* = 32). Of all the filaments carrying the *mcy*TD insertion (*n* = 165) only 6 filaments contained a second mutation (deletion in *mcy*D, insertion into *mcy*EG, *mcy*HA deletion, and insertion into *mcy*A). The filaments carrying the insertion of *mcy*EG (*n* = 60) comprised only two filaments with another mutation (insertion of *mcy*TD and *mcy*EG). Taking into account the probability that one filament carrying an insertion is hit by another insertion/deletion is quite low (only 18 % of all filaments carried two mutations), the co-occurrence between *mcy*HA and *mcy*D deletions was considered remarkable.

When considering each population separately, the ratio of the proportion of neutral MC synthesis mutations vs. MC synthesis-inactivating mutations was more variable. Particularly, in Lake Mondsee, the number of *mcy*D and *mcy*HA deletions was equal to or exceeded the number of MC synthesis neutral mutations (Table 1). On average, the *mcy*D and *mcy*HA partial deletions were more abundant than *mcy*D and *mcy*A insertions. In contrast, in Lake Zürichsee, Hallwilersee, and Ammersee rather few *mcy* gene cluster mutations resulting in the inactivation of MC synthesis were observed. In summary, although the MC synthesis-inactivating mutations were never found to be dominant, spatial and seasonal variability in their proportion occurred.

## Discussion

### Single filament genetics

In this study, using the short-term sonification of filaments, large DNA fragments up to 6 kbp were amplified from single filaments. Assuming a genome size of 5 mega base pairs and four chromosome copies per cell [22], it was calculated that the shortest filaments contained 2.8 pg (440 μm) and the longest filament (6600 μm) contained 42 pg of DNA resulting in 126–1890 *mcy* gene copies (Additional file 1). The influence of length variation among filaments on the PCR result variability was small, suggesting the DNA template amount to be of minor influence. Indeed, it was observed that longer filaments have a stronger tendency to stick to the walls of the reaction tube, and without immersion into PCR buffer they were lost from the PCR analysis. However, we cannot exclude a positive relationship between filament length and the proportion of PCR positive filaments if populations with on average shorter filaments (<1000 μm) would have been included.

This study proposes an alternative method to metagenomics approaches with the aim to elucidate genetic variation on an individual basis. Since in general planktonic cyanobacteria grow clonal, resulting in the formation of macroscopic colonies or filaments, single isolated colonies and filaments can be considered a single genotype [23]. Walsby and co-workers were the first to use DNA obtained from heat-burst single filaments stored in PCR buffer and up to four different PCR amplifications of one or several gene loci were achieved [24]. So far, single colony/filament PCR techniques were applied to amplify relatively short DNA regions (a few hundreds of base pairs). In contrast, genomic approaches on the single cell level have revealed high genetic diversity within populations, e.g., of high-light adapted ecotypes of *Prochlorococcus* [25]. Since even within the cyanobacteria a significant cultivation bias toward a few genera exists, combining single colony isolation with more advanced molecular biological methods holds great potential to analyze the genetic and ecophysiological diversification of cyanobacteria at the individual level.

### Location of *mcy* gene cluster mutations

All the mutations arising from insertions into the *mcy* gene cluster were not randomly distributed but correlated with palindromic RRs of high genetic similarity (Fig. 3). The insertion of ISPlr1 was never found located outside such RRs, suggesting that the insertion of ISPlr1 depends on this type of inverted repeat sequence motif recognition that might be assisted by DNA stem-loop formation. Fewer et al. [10] described non-autonomous mobile miniature inverted-repeat transposable elements (MITEs) to insert into *mcy*D of *Anabaena* isolated from the Baltic Sea. In this study, the inverted RR observed for *Planktothrix* did not show the terminal inverted repeats that are typical for transposable elements [26]. Rather, these RR are similar to REPs that are characterized by a short stretch of high G-C content and have been associated with insertion of transposable elements in many bacteria previously [27]. For the cyanobacteria, REPs have long been used to aid taxonomic classification [28], but their role for genomic mutation and reorganization has been less explored. Notably, the short *mcy*A variant and *rus*A gene inserted into *mcy*TD also contained this RR, suggesting that the insertion of DNA fragments other than IS elements might be favored as well. The RR sequence reported in this study could not be found within *mcy* gene clusters sequenced from other cyanobacteria, i.e., *Anabaena* strain 90 [8] or *Nodularia* [29] using standard BLASTn search algorithm. Only for *Microcystis* the short motif 5′-CCCCAAACCCC-3′ occurred once within *mcy*E (strains PCC7806 and K-139, [6]; [30]) or twice in *mcy*E and *mcy*D (strain NIES 843, [31]). The absence of these *Planktothrix* specific RR in other cyanobacteria fits to the overall conclusion that the *mcy* gene cluster evolved from a cyanobacterial ancestor that occurred two billion years ago and then diversified according to the speciation of the different genera [32]. Consequently, the presence of these RR sequences within the *mcy* gene cluster of *Planktothrix* can explain why the insertion of transposable elements into *mcy* genes are the most common in *Planktothrix* populations and more rare for *Microcystis* and *Anabaena* [33].

### Emergence of the *mcy* gene cluster mutations

In general, no congruency between phylogenetic diversification and a certain mutation was observed (Table 2). A straightforward explanation is that all of these mutations do not provide sufficient selective advantage to lead to their phylogenetic fixation. Still, the question remains as to why those mutants are maintained at relatively high frequency in nature. All of these mutations have been found distributed across the lakes of the Alps, and even the partial deletion of *mcy*D that has been discovered in this study has been detected in all study lakes (R.K. unpublished results). One possible explanation is that the same mutations emerge repeatedly and independently and die off regularly. This speculation is supported through the seasonal variation in abundance of the mutations in Lake Mondsee. It has been argued that the preservation of a certain ability of evolvability is of selective advantage for the total population that has been used as a kind of insurance of genome flexibility in unstable environments [34]. We have preliminary evidence that the activity of ISPlr1 also can be observed *in vivo* using the single filaments of *Planktothrix* strain No110 carrying ISPlr1 insertion within *mcy*D and *mcy*EG-IGS ([9]; C.Q., G.C., R.K. unpublished results). It is tempting to speculate that the varying abundance of MC synthesis mutations is based on variable ISPlr1 activity, which itself might be regulated by environmental conditions.

### Abundance of microcystin synthesis inactivating mutations

The mutations described from single filaments have also been found among isolated clonal strains ([9], R.K., unpublished results). Thus, mutations that inactivate MC synthesis could be differentiated from MC synthesis neutral mutations by strain analysis as described previously [11, 13]. In particular strains carrying the *mcy*EG-IGS insertion (strains SAG6.89, CCAP1459/21, 1459/16, No31/1, 72, 82), as well as strains carrying the insertion between *mcy*T and *mcy*D (CCAP1459/24, No21/2, 64, 241), all produce MC [35]. In contrast strains carrying the ISPlr1 insertion in *mcy*D (No110, 139, 145, 161, 166, 169, 170, 178) or *mcy*A (No40), and strains with a partial deletion of *mcy*HA (No62, 65) or *mcy*D (No130, 137, 194) did not produce MC [9, 13, 35]. Taking all of the filaments together, the number of ISPlr1 inserted into *mcy*EG-IGS exceeded the number of ISPlr1 by far that was inserted directly into *mcy*A or *mcy*D. Since the former does not lead to the inactivation of MC synthesis, while the latter does, it might be speculated that selective pressure is preventing a high frequency of inactive *mcy* genotypes. Frequency-dependent selective pressure has been reported for the coexistence of genotypes in Pseudomonads either carrying plasmids providing mercury detoxification or plasmids free of mercury detoxification genes at a certain range of mercury concentration [36]. Taking into account that MC provides protection against grazers and parasites [37], such frequency dependent selective pressure would be a reasonable scenario that keeps functional *mcy* genotypes as the dominant part of the *Planktothrix* sp. population in Lake Mondsee since its first observation in 2002 [13].

On the other hand, among the population from Lake Mondsee, the number of *mcy*D and *mcy*HA deletions outweighed the number of *mcy*EG-IGS ISPlr1 genotypes at least at two sampling dates. At present, it is not known as to whether the imprecise excision of ISPlr1 can cause the partial deletions observed within *mcy*D and *mcy*HA. For many years, transposons Tn5, Tn7, Tn10 and bacteriophage Mu have been described to excise imprecisely from their point of insertion with the concomitant creation of deletions [38]. Among the 55 filaments carrying the partial deletion of *mcy*D, none carried an insertion of ISPlr1 into *mcy*D. In contrast, the partial deletion of *mcy*HA was only observed subsequent to the recombination of the short *mcy*A variant. This *mcy*A short variant, however, carried the same RR sequence as has been found at the ISPlr1 insertion sites, and it seems possible that an imprecise excision of ISPlr1 finally leads to both partial deletions in *mcy*D and *mcy*A. Accordingly, Vasas et al. [39] reported the occurrence of a partial deletion within the *mcy*EG-IGS region of a *P. rubescens* strain isolated from a gravel pit pond in Hungary. In the future, more quantitative data will be needed to find out whether short-term shifts in the abundance of mutations occur and what the factors are that are leading to it.

## Conclusions

By observing recombination processes within the *mcy* gene cluster of hundreds of single filaments in real-time, we conclude that insertion events occurring within the *mcy* gene cluster of *Planktothrix* are predictable due to the dependence on repetitive nucleotide sequence motifs, which represent REPs that are described as IS element insertion sites from other bacterial phyla. The higher abundance of those REPs within the *mcy* gene cluster of *Planktothrix* but not in other genera *Microcystis* and *Anabaena* can explain the more frequent mutation of the *mcy* gene cluster in *Planktothrix* when compared with other genera. Although the MC synthesis inactive genotypes were not observed to be dominant, we found evidence that under certain conditions a higher proportion of MC synthesis inactive *mcy* genotypes occurred, which might be caused by the increased activity of ISPlr1.

## Methods

### Study area and sampling

Five lakes located in the Alps, Mondsee (47°49′N, 13°22′E), Wörthersee (46°37′N, 14°07′E), Zürichsee (47°15′N, 08°38′E), Hallwilersee (47°17′N, 08°12′E), Ammersee (47°59′N, 11°07′E) were sampled for red-pigmented *P. rubescens* filaments by pulling a plankton net (30 μm mesh size) from a depth of 20 m to the surface from a boat in the middle of the lake (Table 1). The lakes are deep, physically stratified, and have been shown to inhabit red-pigmented *Planktothrix* during the summer either seasonally (Ammersee) or perennially (Mondsee, Wörthersee, Zürichsee, Hallwilersee). No specific permissions were required for these locations/activities, as in all three countries (Austria, Germany, Switzerland) water quality analyses are free of permission in public waterbodies. In addition the field study did not involve endangered species.

### Filament isolation

Individual *P. rubescens* filaments were picked randomly from diluted samples under a dissecting microscope as described [13]. Each filament was washed three times by subsequent transfers between drops of BG11 medium [40], measured in length and finally transferred to a 0.5 ml Eppendorf tube containing 10 μl of sterile Millipore water and stored at −20 °C.

### DNA extraction

Single *Planktothrix* filaments were ultrasonified using a sonifier cell disruptor equipped with a microtip (Branson, Danbury, Connecticut, USA) under optimized conditions (see Additional file 3). The microtip was washed with 10 % (v/v) H_2_O_2_ between individual samples. The quality of the obtained DNA fragments was tested with an Agilent Bio-analyzer 2100 (Agilent Technologies, Palo Alto, CA) following manufacturer’s instructions. In a pilot experiment filaments were isolated from *P. rubescens* strain No3 and were sonified in different concentrations of 100, 50 and 10 filaments μl^−1^ (in 10 μl, 15 % ultrasound strength, 1 s) to analyze the fragment size distribution of the obtained DNA. Independent from filament concentration a major peak of DNA elution was observed at 6000 bp, indicating that DNA templates of appropriate size for subsequent PCR amplification were available. As expected, DNA concentration and height of the major peak increased linearly with the number of sonified filaments (Additional file 3). In addition, the variable intensity of sonification (10, 15, 20, 25, 30, 35 and 40 % of total output, 100 filaments in 10 μl, 1 s) revealed an overall increase in DNA concentration. In contrast, the size of the DNA and the major peak height decreased indicating the increased fragmentation of the DNA (Additional file 3). Analogously, the DNA concentration increased with longer sonification time (1, 20, 30, 90, 180, 270 and 450 s, 100 filaments, in 100 μl, 15 % ultrasound strength), (Additional file 3). However, both DNA size and the major peak height decreased suggesting that DNA became increasingly fragmented with sonification time. Thus, in order to guarantee maximum DNA size, a low sonification intensity (15 %) and a short sonification time (1 s) were used during further analyses.

### DNA storage time

To analyze the storage time of extracted DNA isolated from individual filaments, DNA was both stored at −20 °C in Millipore water and in Phire Hot Start Polymerase Plant PCR dilution buffer (Finnzymes, Espoo, Finland). No PCR products were obtained from DNA extracted from filaments stored in Millipore water for more than two days (Additional file 4). In contrast, DNA extracted from filaments but stored in PCR dilution buffer consistently revealed PCR products during a storage time until 9 months (data not shown). Consequently, DNA stored in PCR dilution buffer was used for all further application.

In general, 16 primer pairs (F*mcy*amplify fragments of 3.5 kbp of the *mcy* gene cluster without interruption (see below). Pilot tests showed that using the Phire Hot start II DNA polymerase (Finnzymes, Espoo, Finland) following manufacturer’s instructions, PCR products ranging from 500 bp to 9 kbp could be obtained from one single filament. In order to determine the maximum of PCR assays possible for each filament sample, DNA extracted from individual filaments was diluted with Phire Hot Start Polymerase PCR buffer 1-, 2-, 4-, 8-, and 16-fold. Using both the PC-IGS and 16S rDNA primer pairs (see below), PCR amplification was achieved until 16-fold dilution (Additional file 5).

### PCR amplification

In order to confirm the presence of *Planktothrix* DNA after the sonification of each individual filament, the IGS between *psa*A and *psa*B was amplified (Additional file 6). The PCR was performed using Dream Taq polymerase (Thermo Scientific, Fermentas, St. Leon Rot, Germany) in 10 μl, containing 1 μl of Dream Taq PCR buffer (10×), 0.4 μl of MgCl_2_ (50 mM), 0.3 μl of dNTPs (10 mM each), 0.3 μl of each primer (10 pmol μl^−1^), 0.05 μl of polymerase, 6.65 μl sterile Millipore water and 1.0 μl DNA template. The PCR thermal cycling protocol included an initial denaturation step at 94 °C for 3 min, followed by 35 cycles (denaturation at 94 °C for 30 s, annealing at 60 °C for 30 s, elongation at 72 °C for 30 s), and a final elongation step at 72 °C for 1 min.

In order to detect all the potential deletions and insertions within the entire *mcy* gene cluster, 16 primer pairs (F*mcy*1 - 16) were designed and used to amplify fragments of 3.5 kbp without interruption (Additional file 6). Individual filament DNA samples were diluted 5-fold resulting in 50 μl of DNA template per filament in total. The PCR amplifications were performed in reaction mixtures of 10 μl, containing 2 μl of PCR reaction buffer (5×), 0.2 μl of dNTPs (Kapa Biosystems, Woburn, MA, USA), 0.5 μl of each primer (10 pmol μl^−1^), 0.2 μl of Phire Hot start II DNA polymerase, 5.6 μl sterile Millipore water and 1 μl DNA template. The PCR thermal cycling protocol included an initial denaturation step at 98 °C for 30 s, followed by 40 cycles (denaturation at 98 °C for 5 s, annealing at 60 °C for 20 s, elongation at 72 °C for 70 s) and final elongation (72 °C, 1 min). The mutations were detected via the size difference of the PCR products during ethidium bromide stained gel electrophoresis as compared to the PCR products obtained from the reference strain *P. agardhii* NIVA-CYA126/8 (AJ441056) [7]. If no PCR product was obtained, shorter PCR products (1.75 kbp, 35 s annealing) were amplified by combining a primer from the set F*mcy*1 - 16 with the corresponding primer from the set F*mcy*m1 - 16 (Additional file 6), which were located in the middle of each 3.5 kbp fragment. In order to identify insertions (i.e., the *mcy*TD insertion and *mcy*EG insertion), specific primer pairs were used, which consisted of one primer binding to a locus within the *mcy* gene cluster and the second primer binding to a locus within the insert. For the sequencing of selected PCR products, gene fragments were amplified using the 3′–5′exonuclease-containing Phusion polymerase (Finnzymes, Espoo, Finland). The PCR amplifications were performed in reaction mixtures of 25 μl, containing 5 μl of Phusion GC reaction buffer, 0.5 μl of dNTPs (10 mM of each, Kapa), 1.25 μl of each primer (10 pmol μl^−1^), 0.25 μl of Phusion DNA polymerase, 15.8 μl sterile Millipore water and 1 μl DNA template. The PCR thermal cycling protocol was identical to the Phire Hot start II DNA polymerase.

### Phylogenetic analysis

In order to assign individual filaments to *Planktothrix* phylogenetic groups, the IGS of *cpc*BA (the intergenic spacer region between phycocyanin B and phycocyanin A protein) and *rbc*LX (the intergenic spacer region between the large subunit of the ribulose bisphosphate carboxylase/oxygenase and *rbc*X) were amplified and sequenced (Additional file 6). The oligonucleotides used to amplify the *cpc*BA and *rbc*LX locus were designed previously [13], [16]. The PCR thermal cycling protocol included an initial denaturation step at 98 °C for 30 s, followed by 40 cycles (98 °C for 5 s, 66 °C for 20 s, 72 °C for 30 s) and final elongation (72 °C, 1 min). PCR products were purified and sequenced by standard automated fluorescence techniques (Applied Biosystems, Weiterstadt, Germany). All sequence data have been submitted to the DDBJ/EMBL/GenBank databases under accession numbers KP315865 - 73 (*cpc*BA, 475 bp), KP315874 - 80 (*rbc*LX, 390 bp), KP315862 - 64 (the insertion into the intergenic region of *mcy*TD, 1194 bp), KP315861 (the flanking region of the inserted short *mcy*A variant, 447 and 428 bp), KP710231 (the partial deletion of *mcy*D, 1964 bp), (Table 2).

### Availability of supporting data

For nucleotide acid sequences submitted to the DDBJ/EMBL/GenBank databases under accession numbers KP315861-80, KP710231 see text. The data supporting the results of this article are included in Additional files 1, 2, 3, 4, 5, 6 and 7.

## Additional files

Additional file 1:
**Relationship between** ***Planktothrix*** **filament length and cell number and length of** ***Planktothrix*** **filaments as compared between samples found PCR negative or PCR positive.** (DOCX 17 kb) Additional file 2:
**Summary of repetitive regions (RR) and associated mutations through ISPlr1 and RusA occurring within the** ***Planktothrix mcy*** **gene cluster.** (DOCX 13 kb) Additional file 3:
**Measures of genomic DNA isolated from** ***Planktothrix*** **filaments using a cell disruptor sonifier.** (DOCX 21 kb) Additional file 4:
**Effect of storage time (−20 °C) of DNA extracted from sonified** ***Planktothrix*** **filaments in Millipore water on PCR amplification of the PC-IGS region.** (DOCX 766 kb) Additional file 5:
**Amplification of PCR products from DNA extracted from one single** ***Planktothrix*** **filament and diluted 1-, 2-, 4-, 8-, and 16-fold.** (DOCX 916 kb) Additional file 6:
**Oligonucleotides used in the present study.** (DOCX 19 kb) Additional file 7:
**Occurrence of mutations in individual** ***Planktothrix*** **filaments (Excel spreadsheet).** (XLSX 74 kb)

## Abbreviations

MC: Microcystin
REPs: repetitive extragenic palindromic DNA sequences
NRPS: nonribosomal peptide synthetase
IGS: intergenic spacer region
IS element: Insertion sequence element
ISPlr1: IS element in *P. rubescens*
IRL: IRR, inverse repeat left, inverse repeat right
DR: direct repeat
RRs: GC rich repetitive regions
MITE: non-autonomous mobile miniature inverted-repeat transposable element
