# Supplementary material for: Emergence of nontoxic mutants as revealed by single filament analysis in bloom-forming cyanobacteria of the genus Planktothrix
Source: BMC Microbiol. 2016 Feb 25;16:23. doi: 10.1186/s12866-016-0639-1 (PMC4766695; doi:10.1186/s12866-016-0639-1)
Supplement: Additional file 1: — Relationship between Planktothrix filament length and cell number and length of Planktothrix filaments as compared between samples found PCR negative or PCR positive. (DOCX 17 kb) [file 12866_2016_639_MOESM1_ESM.docx]

**Additional File 1.** (A) Relationship between *Planktothrix* filament length (27 Sep 2011, Mondsee) and cell number (n = 30): y = 1.42 + 0.31 × x, where y is cell number and x in filament length in µm). (B) Length of *Planktothrix* filaments as compared between samples found PCR negative (n = 86) or PCR positive (n = 828).
