# Supplementary material for: Emergence of nontoxic mutants as revealed by single filament analysis in bloom-forming cyanobacteria of the genus Planktothrix
Source: BMC Microbiol. 2016 Feb 25;16:23. doi: 10.1186/s12866-016-0639-1 (PMC4766695; doi:10.1186/s12866-016-0639-1)
Supplement: Additional file 2: — Summary of repetitive regions (RR) and associated mutations through ISPlr1 and RusA occurring within the Planktothrix mcy gene cluster. (DOCX 13 kb) [file 12866_2016_639_MOESM2_ESM.docx]

**Additional File 2.** Summary of repetitive regions (RR) and associated mutations through ISPlr1 and RusA occurring within the *Planktothrix mcy* gene cluster., n/a… not applicable.

| **Name** | **Length** | **Position** | **Locus** | **Characteristics** |
| --- | --- | --- | --- | --- |
|  |  |  |  |  |
| RR No1 | 43 | 11,896^1^ | *mcy*D | One insertion site of ISPlr1 |
| RR No2 | 43 | 23,779^1^ | IGS of *mcy*EG | Two insertion sites of ISPlr1 |
| RR No3 | 44 | 38,853^1^ | *mcy*A | One insertion site of ISPlr1 |
| RR No4 | 44 | 25^2^ | *mcy*TD insertion | within the insertion RusA |
| RR No5 | 45 | 604^3^ | *mcy*A(short variant) | Within the inserted *mcy*AA1-adenylation domain |
| RR No6 | 45 | 53,747^1^ | 121 bp downstream of *mcy*J | No mutations observed |
| RR No7 | 45 | n/a | 622 bp upstream of *mcy*T | Not analyzed |

^1^ according to access. No. AJ441056

^2^ according to access. No. KP315862-64

^3^ according to access. No. AJ749265
