# Supplementary material for: Emergence of nontoxic mutants as revealed by single filament analysis in bloom-forming cyanobacteria of the genus Planktothrix
Source: BMC Microbiol. 2016 Feb 25;16:23. doi: 10.1186/s12866-016-0639-1 (PMC4766695; doi:10.1186/s12866-016-0639-1)
Supplement: Additional file 3: — Measures of genomic DNA isolated from Planktothrix filaments using a cell disruptor sonifier. (DOCX 21 kb) [file 12866_2016_639_MOESM3_ESM.docx]

**Additional File 3.** Average ± SD of total peak area, concentration and max. peak height of genomic DNA isolated from *Planktothrix* filaments using a cell disruptor sonifier as monitored by the Agilent Bio-analyzer 2100. DNA yield in dependence on (A) the number of filaments; (B) sonification amplitude; (C) sonification time.
