# Supplementary material for: Emergence of nontoxic mutants as revealed by single filament analysis in bloom-forming cyanobacteria of the genus Planktothrix
Source: BMC Microbiol. 2016 Feb 25;16:23. doi: 10.1186/s12866-016-0639-1 (PMC4766695; doi:10.1186/s12866-016-0639-1)
Supplement: Additional file 4: — Effect of storage time (−20 °C) of DNA extracted from sonified Planktothrix filaments in Millipore water on PCR amplification of the PC-IGS region. (DOCX 766 kb) [file 12866_2016_639_MOESM4_ESM.docx]

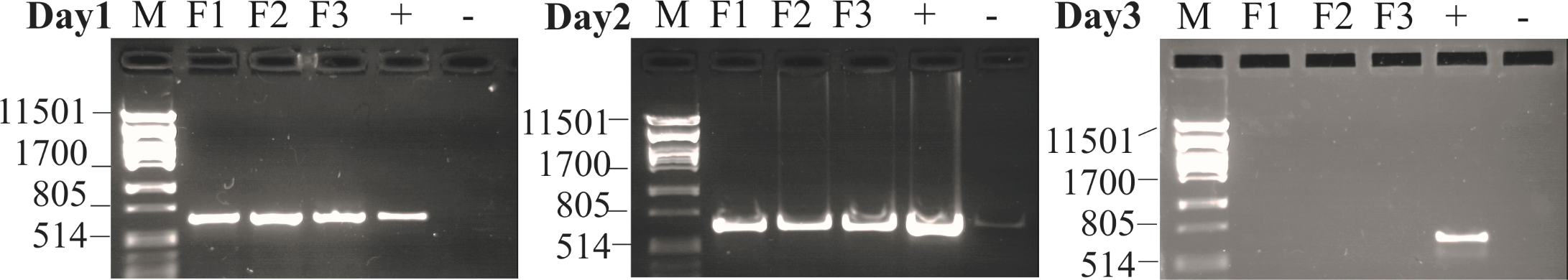


**Additional File 4.** Effect of storage time (-20°C) on PCR amplification of the PC-IGS region from three sonified *Planktothrix* filaments (F1, F2, F3) during three subsequent days. M, PstI lambda DNA size marker. Positive control was amplified from *P. agardhii* NIVA-CYA126/8 (AJ441056).
