# Supplementary material for: Emergence of nontoxic mutants as revealed by single filament analysis in bloom-forming cyanobacteria of the genus Planktothrix
Source: BMC Microbiol. 2016 Feb 25;16:23. doi: 10.1186/s12866-016-0639-1 (PMC4766695; doi:10.1186/s12866-016-0639-1)
Supplement: Additional file 5: — Amplification of PCR products from DNA extracted from one single Planktothrix filament and diluted 1-, 2-, 4-, 8-, and 16-fold. (DOCX 916 kb) [file 12866_2016_639_MOESM5_ESM.docx]

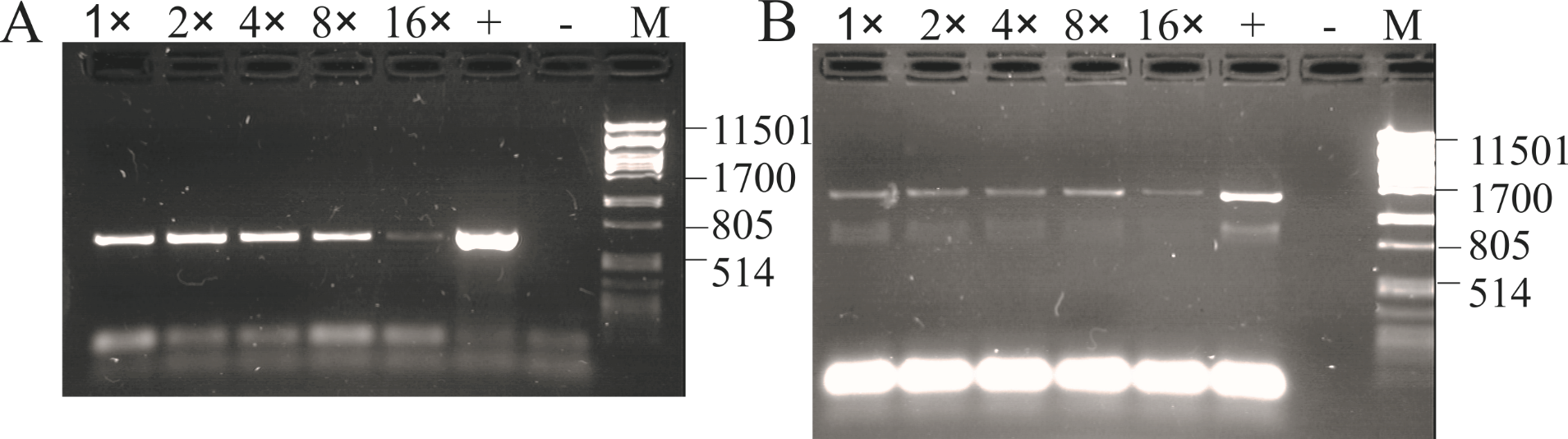


**Additional File 5.** Amplification of PCR products from (A) *psa*BA-IGS or (B) 16S rDNA gene locus from DNA extracted from one single *Planktothrix* filament and diluted 1-, 2-, 4-, 8-, and 16-fold using the Phire Hot Start Polymerase PCR buffer. M, PstI lambda DNA size marker. Positive control was amplified from *P. agardhii* NIVA-CYA126/8 (AJ441056).
