# Supplementary material for: Emergence of nontoxic mutants as revealed by single filament analysis in bloom-forming cyanobacteria of the genus Planktothrix
Source: BMC Microbiol. 2016 Feb 25;16:23. doi: 10.1186/s12866-016-0639-1 (PMC4766695; doi:10.1186/s12866-016-0639-1)
Supplement: Additional file 6: — Oligonucleotides used in the present study. (DOCX 19 kb) [file 12866_2016_639_MOESM6_ESM.docx]

**Additional File 6.** Oligonucleotides used in the present study.

|  | Forward Primer | | | | Reverse Primer | | | | PCR  product  length  (bp) |
| --- | --- | --- | --- | --- | --- | --- | --- | --- | --- |
| Gene locus | Name | Sequence (5’-3’) | bp number^1^ | Tm (°C) | Name | Sequence | bp number^1^ | Tm (°C) |  |
| Entry PCR | | |  | | | | | | |
| *psa*A, *psa*B | *Psa*fwd^2^ | GGGTGGTACTTGCCAAGTCTCT | - | 58 | *Psa*rev^2^ | CGACGTGTTGTCGGGTCTT | - | 58 | 669 |
| 16SrDNA-ITS | CYA359F^3^ | GGGGAATTTTCCGCAATGGG | - | 67.5 | 23S30R^3^ | CTTCGCCTCTGTGTGCCTAGGT | - | 65.1 | 1673 |
| Screening of the entire *mcy* gene cluster in steps of 3.5 kbp | | |  | | | | | | |
| *mcy*TD | F*mcy*1+ | ATTTTCCCAAGCATTCTAGG | 481-500 | 60.06 | F*mcy*1- | TCCAATTTCTAGGAAGATTTG | 3674-3694 | 60.90 | 3213 |
| *mcy*D | F*mcy*2+ | AGCAGTTAATAATGATGGCG | 2314-2333 | 59.72 | F*mcy*2- | GGCTGGGTTCAATAATATTAA | 5922-5942 | 60.98 | 3628 |
| *mcy*D | F*mcy*3+ | GCCACAGCAGATATTCAAAA | 5696-5715 | 60.77 | F*mcy*3- | TGAATATTGCGATCCTTGTC | 9333-9352 | 60.90 | 3656 |
| *mcy*D | F*mcy*4+ | CCAAAAAGGTGAGGATTCTC | 9253-9272 | 60.59 | F*mcy*4- | ATCTGTTCAATTCCTTGAGAT | 12775-12795 | 60.27 | 4542 |
| *mcy*DE | F*mcy*5+ | TTCCGCCAAATTATTACAGA | 12715-12734 | 60.06 | F*mcy*5- | ATGGGTAAGGTTTGCTTGAT | 16409-16428 | 60.35 | 3713 |
| *mcy*E | F*mcy*6+ | TCTCAATTACAACCTATTGCA | 16313-16333 | 60.87 | F*mcy*6- | AATTCGGCTTAAAAAAGCTG | 19890-19909 | 60.35 | 3576 |
| *mcy*E | F*mcy*7+ | CAGCTTTTTTAAGCCGAATT | 19890-19909 | 60.35 | F*mcy*7- | TTCAAGTATAATTCTCCTTCCTC | 23441- 23463 | 60.60 | 3554 |
| *mcy*EG | F*mcy*8+ | AACTCCATTGACCCAGAGAT | 23384-23403 | 60.50 | F*mcy*8- | ATTAAACCCACAATACCGGA | 27032-27043 | 60.95 | 3640 |
| *mcy*G | F*mcy*9+ | AATATCGGACATATGCAAATT | 27003-27023 | 60.94 | F*mcy*9- | AGTAAATCAGCAATATTTTGAGTAA | 30481-30505 | 60.64 | 3502 |
| *mcy*GHA | F*mcy*10+ | GCGCCGATATTACTCAAAAT | 30472-30491 | 60.94 | F*mcy*10- | GAATCGGCAAAAAATTAAGC | 34041-34060 | 60.54 | 3588 |
| *mcy*A | F*mcy*11+ | GGAGCCTTAAATATAGAAACATC | 34013-34035 | 60.13 | F*mcy*11- | GATTCCAAACAATAGCGACA | 39010- 39029 | 60.63 | 5016 |
| *mcy*AB | F*mcy*12+ | TTTGAAATCCAAGTTGAACG | 38977-38997 | 60.63 | F*mcy*12- | AATTTGTAAAACTTCCCCATA | 42681-42701 | 60.43 | 3724 |
| *mcy*B | F*mcy*13+ | TATGGGGAAGTTTTACAAATT | 42681-42701 | 60.15 | F*mcy*13- | TGGCAATATCCAACTCAGAT | 46081-46100 | 60.06 | 3419 |
| *mcy*BC | F*mcy*14+ | ATCTGAGTTGGATATTGCCA | 46081-46100 | 60.06 | F*mcy*14- | TGGATGATTTCTTCCAGA | 49580-49597 | 60.16 | 3516 |
| *mcy*CJ | F*mcy*15+ | TTCTCAAGCAGCTTCATATAC | 49411-49431 | 60.90 | F*mcy*15- | AACTCTTTCTTGAGCAATTTC | 53086-53106 | 60.31 | 3695 |
| *mcy*J | F*mcy*16+ | AAGCTGGAGATCAAGTTTTAG | 52970-52990 | 60.34 | F*mcy*16-^4^ | TTTGTGTGTCTTGGTTAGGG | 56573-56593 | 60.56 | 3624 |
| Screening of the entire *mcy* gene cluster in shorter intervals (1.75 kbp) | | |  | | | | | | |
| *mcy*D | F*mcy*m1+ | ATCAGATGTTAGCCTCCGAT | 2163-2182 | 60.61 | F*mcy*m1- | ATCGGAGGCTAACATCTGAT | 2163-2182 | 60.61 | - |
| *mcy*D | F*mcy*m2+ | CCTTACTAGGTGATCGTTTTC | 3984-4004 | 60.45 | F*mcy*m2- | GAAAACGATCACCTAGTAAGG | 3984-4004 | 60.45 | - |
| *mcy*D | F*mcy*m3+ | AAATGATGAAACTATTGCTCC | 7468-7488 | 60.78 | F*mcy*m3- | GGAGCAATAGTTTCATCATTT | 7468-7488 | 60.78 | - |
| *mcy*D | F*mcy*m4+ | TAGAATTATTCCGAGATCCTG | 11061-11081 | 60.87 | F*mcy*m4- | CAGGATCTCGGAATAATTCTA | 11061-11081 | 60.87 | - |
| *mcy*E | F*mcy*m5+ | CAACCCCTTCCTAGTCATCT | 14576-14595 | 60.09 | F*mcy*m5- | AGATGACTAGGAAGGGGTTG | 14576-14595 | 60.09 | - |
| *mcy*E | F*mcy*m6+ | ATATTTTCCGGCTCCTATCA | 18149-18168 | 60.40 | F*mcy*m6- | TGATAGGAGCCGGAAAATAT | 18149-18168 | 60.40 | - |
| *mcy*E | F*mcy*m7+ | CCGGTGTGATTGAATTTATG | 21657-21676 | 60.74 | F*mcy*m7- | CATAAATTCAATCACACCGG | 21657-21676 | 60.74 | - |
| *mcy*G | F*mcy*m8+ | AGCAATGGGTAAAAGTCGTT | 25417-25436 | 60.52 | F*mcy*m8- | AACGACTTTTACCCATTGCT | 25417-25436 | 60.52 | - |
| *mcy*G | F*mcy*m9+ | AAACGGTATCGGCTATTGTT | 28735-28754 | 60.36 | F*mcy*m9- | AACAATAGCCGATACCGTTT | 28735-28754 | 60.36 | - |
| *mcy*H | F*mcy*m10+ | GTTTGGGGCTGTTGTTAATT | 32204-82436 | 60.75 | F*mcy*m10- | AATTAACAACAGCCCCAAAC | 32204-82436 | 60.75 | - |
| *mcy*A | F*mcy*m11+ | TAGGCATGATCTTCCACAGT | 37629-37648 | 60.25 | F*mcy*m11- | ACTGTGGAAGATCATGCCTA | 37629-37648 | 60.25 | - |
| *mcy*A | F*mcy*m12+ | ACATAGATTCTATCGGGGATA | 40701-40721 | 60.11 | F*mcy*m12- | TATCCCCGATAGAATCTATGT | 40701-40721 | 60.11 | - |
| *mcy*B | F*mcy*m13+ | TTCTCTGCTGCGACTTTTAC | 44351-44370 | 60.30 | F*mcy*m13- | GTAAAAGTCGCAGCAGAGAA | 44351-44370 | 60.30 | - |
| *mcy*B | F*mcy*m14+ | AATATTTCCTATGCGCGAGT | 47799-47818 | 60.59 | F*mcy*m14- | ACTCGCGCATAGGAAATATT | 47799-47818 | 60.59 | - |
| *mcy*C | F*mcy*m15+ | TGCCAATACCCAAATTTATAT | 51094-51114 | 60.88 | F*mcy*m15- | ATATAAATTTGGGTATTGGCA | 51094-51114 | 60.88 | - |
| IS element^5^ | F*mcy*m16+ | GGGATGGAAAATCAACAATT | 54637-54656 | 60.53 | F*mcy*m16- | AATTGTTGATTTTCCATCCC | 54637-54656 | 60.53 | - |
| Identification of specific insertions | |  | | | | | | | |
| *mcy*TD | TD5+ | TACCCAGGAGTTAGCAGT | 1187-1204 | 59.90 | TD5- | TAGCCCAAGCAGTAGAAAAA | - | 59.60 | 624 |
| *mcy*TD | TD3+ | GAAGACCAGTATTTTTCCGC | - | 60.60 | TD3- | AATCGCTATGGGTTCACTTT | 1512-1527 | 60.50 | 396 |
| *mcy*EG | EG5+ | AACTCCATTGACCCAGAGAT | 23384-23403 | 60.50 | EG5- | AAAAAAATGGTGATCGCTCT | - | 60.60 | 544/556 |
| *mcy*EG | EG3+ | GGAAGAAGGTGGTTAGGAAA | - | 60.10 | EG3- | AAAGTGTCCGCCAATTATCT | 23939-23958 | 60.50 | 412/400 |
| Phylogenetic analysis |  | | | | | | | | |
| *rbc*LX | *rbc*fwd^2^ | TGTTCAAGCGCGTAACGAAG | - | 66.50 | *rbc*rev | CCAGGGTTAGTTTCCCATAGTTGAT | - | 66.00 | 382 |
| *cpc*BA | *cpc*BA + | TTCTGAAATCGCCGGCTACT | - | 66.15 | *cpc*BA - | TCCATAGGGCCTGTACCACC | - | 66.24 | 470 |

^1^ according to *P. agardhii* NIVA-CYA126/8 (AJ441056), reference [1], ^2^ Primers from reference [2], ^3^ see reference [3]; ^4^ F*mcy*16- binds 2,948 bp downstram of *mcy*J as revealed from NIVA-CYA126/8 genome (Access No. ASAK01000000); ^5^IS element group I flanking the *mcy* gene cluster [4].

References

1. Christiansen G, Fastner J, Erhard M, Börner T, Dittmann E: **Microcystin biosynthesis in *Planktothrix*: genes, evolution, and manipulation**. *J Bacteriol* 2003, **185**(2):564-572.

2. Christiansen G, Molitor C, Philmus B, Kurmayer R: **Nontoxic strains of cyanobacteria are the result of major gene deletion events induced by a transposable element**. *Mol Biol Evol* 2008, **25**(8):1695-1704.

3. Taton A, Grubisic S, Brambilla E, De Wit R, Wilmotte A: **Cyanobacterial diversity in natural and artificial microbial mats of Lake Fryxell (McMurdo Dry Valleys, Antarctica): a morphological and molecular approach**. *Appl Environ Microbiol* 2003, **69**(9):5157-5169.

4. Christiansen G, Goesmann A, Kurmayer R: **Elucidation of insertion elements carried on plasmids and *in vitro* construction of shuttle vectors from the toxic cyanobacterium *Planktothrix***. *Appl Environ Microbiol* 2014, **80**(16):4887-4897.
